# Extending the global worm index and its links to human development and child education

**DOI:** 10.1371/journal.pntd.0006322

**Published:** 2018-06-21

**Authors:** SuJin Kang, Ashish Damania, M. Farhan Majid, Peter J. Hotez

**Affiliations:** 1 Center for Health & Biosciences, James A. Baker III Institute for Public Policy, Rice University, Houston, Texas, United States of America; 2 Department of Pediatrics and Molecular Virology and Microbiology, National School of Tropical Medicine, Baylor College of Medicine, Houston, Texas, United States of America; 3 Department of Biology, Baylor University, Waco, Texas, United States of America; Swiss Tropical and Public Health Institute, SWITZERLAND

## Introduction

In 2015, we reported on a new national “worm index” for human development using data released by the World Health Organization (WHO) for the total number of school-aged children requiring mass drug administration (MDA) to treat their intestinal helminth infections and schistosomiasis, together with the population requiring MDA for lymphatic filariasis (LF) [1]. The worm index was calculated by adding these 3 populations at risk and then dividing it by the total population [1]. We found that for the 25 most populated nations, there was a strong inverse association between the national worm index and its human development index (HDI), a summary metric that incorporates living standards, educational attainment, and health [1]. We subsequently found a similar inverse relationship for the world’s Muslim-majority countries belonging to the Organization of Islamic Cooperation (OIC) [2].

Our finding that high national worm indices link to low human development generates a number of hypotheses regarding how human helminth infections adversely affect child development, overall health, and even economic development. Including all intestinal helminth infections, schistosomiasis, and LF also helps to evaluate global effects. For example, many Asian nations might have high worm indices due to widespread intestinal helminth infections and LF, whereas in many African countries, schistosomiasis also contributes greatly to calculating national worm indices [1, 2]. Employing the worm index also presents an opportunity to evaluate how integrated MDA for intestinal helminth infections, schistosomiasis, and LF could both reduce worm indices and translate into health and economic gains as an approach towards sustainable development [1].

Here, we extend our earlier findings by calculating worm indices for all 191 countries for which there are available data. We also calculate the worm index in 2 different ways, using both the methods described earlier that rely on WHO data (now with the most recently reported data in 2016) [1, 2], as well as newly released data from the Institute for Health Metrics and Evaluation (IHME) for the Global Burden of Disease Study (GBD) 2016 [3]. It should be noted that WHO data provides the estimated number of school-aged children (intestinal helminth infections and schistosomiasis) or adults (LF) requiring preventive chemotherapy (PC) for endemic countries, whereas GBD data provides the prevalence of each of the intestinal helminth infections (e.g., ascariasis, trichuriasis, hookworm disease, schistosomiasis, and LF) in every country.

Using either approach, we confirm a strong inverse association between worm indices and HDIs. In addition, we observed especially strong associations between worm indices and low educational attainment, years of schooling, and school completion. Our findings suggest that helminth control and elimination might comprise an essential element of global efforts to improve childhood education.

## Impact of worm indices on human development and education indicators

Table 1 displays the descriptive statistics for 2 different worm indices, including the WHO Parasite Control and Transmission (PCT) database [4] and the GBD 2016 from the IHME website [5], respectively, as well as HDI [6], and 6 indicators of education from either the United Nations Development Programme (UNDP) [6] or the World Bank (WB) [7].

**Table 1:** Descriptive statistics for worm index with human development and education analysis.

Variables	*N*	Mean or percentage	SD	Minimum	Maximum
WHO worm index (2016)	163	0.249	0.355	0	1.414
GBD worm index (2016)	191	0.045	0.057	0	0.226
HDI (UNDP, 2015)	182	0.695	0.156	0.352	0.949
Mean years of schooling (UNDP, 2015) Persistence to last grade of primary (WB, 2014)					
Female	74	85.67%		21.63	100
Male	74	84.38%		21.09	100
Repeaters in primary education (WB, 2015)					
Female	110	3.64%		0	22.99
Male	110	4.31%		0	22.22
Progression to secondary school (WB, 2015)					
Female	95	92.23%		52.82	100
Male	95	92.22%		54.98	100
Net enrollment rate, secondary (WB, 2015)	88	73.1%		17.18	99.66
Mean years of schooling (UNDP, 2015)	181	8.278	3.105	1.4	13.4

Abbreviations: SD, standard deviation; GBD, Global Burden of Disease; HDI, human development index; WB, World Bank; UNDP, United Nations Development Programme.

The most recent HDI and mean years of schooling are from the UNDP data 2015 [6]. HDI measures average achievement in 3 basic dimensions of human development: a long and healthy life, knowledge, and a decent standard of living. Mean years of schooling refers to the average number of years of education received by people aged 25 and older.

From the WB, we examined 3 educational parameters, which also compared female and male students [7]. First, persistence to the last grade of primary refers to “the percentage of children enrolled in the first grade of primary school who eventually reach the last grade of primary education,” [7] using the most recent data available from 2014. Next, repeaters in primary education indicate the percentage of students enrolled in the same grade as in the previous year (2015 data) [7], while progression to secondary school refers to “the number of new entrants to the first grade of secondary school in a given year as a percentage of the number of students enrolled in the final grade of primary school in the previous year” [7] (also 2015 data). Finally, the net enrollment rate to secondary also comes from 2015 data. It refers to “the number of students enrolled who are of the official age group for secondary education divided by the population for the same age group” [7]. To estimate coefficients of the association between worm index and each outcome measure, we used Ordinary Least Square (OLS) bivariate regression analysis.

Table 2 demonstrates the results for bivariate regression analysis. Both of the worm indices from WHO and GBD 2016 exhibit strong and negative associations with all developmental and educational measures (p < .001). It was found that for every standard deviation (SD) unit increases in worm index, the HDI decreases by about 0.731 (WHO) and 0.700 (GBD) SD units, respectively. Results on the association between worm indices and indicators of education show that 1 SD increase in worm index decreases the net enrollment rate to secondary school by about 0.669 (WHO) and 0.639 (GBD) SD units. The average years of schooling also shows similar results. Each SD unit decreases by about 0.667 (WHO) and 0.596 (GBD). Lastly, the analyses of the gender variability between worm indices and education indicators find that first, persistence to the last grade of primary education shows a discrepancy between WHO and GBD data results. For WHO data, worm indices have a slightly more negative impact on female children, whereas for GBD data, the negative outcome is greater for male children. However, repeaters in primary school and progression to secondary education show that worm indices in both WHO and GBD data exhibit greater negative impacts on female children than male. Thus, the overall results indicate that worm indices might exhibit a more negative impact on female children’s educational attainment. However, the Wilcoxon rank-sum test results show that the gender difference on these outcomes are not statistically significant.

**Table 2:** Standardized coefficients (Beta) from the bivariate regression of child development and education on worm index.

	HDI	Net enrollment rate to secondary	Mean years of schooling	Persistence to last grade of Primary		Repeaters in primary school		Progression to secondary education	
				Female	Male	Female	Male	Female	Male
WHO worm index	−0.731	−0.669	−0.667	−0.697	−0.685	0.655	0.622	−0.596	−0.583
GBD worm index	−0.700	−0.639	−0.596	−0.695	−0.725	0.674	0.636	−0.498	−0.491

*P*-values < 0.001

Abbreviations: HDI, human development index; GBD, Global Burden of Disease; WB, World Bank.

Sources: WHO; GBD; WB.

## Countries and regions highly affected by worm indices

**Fig 1:**
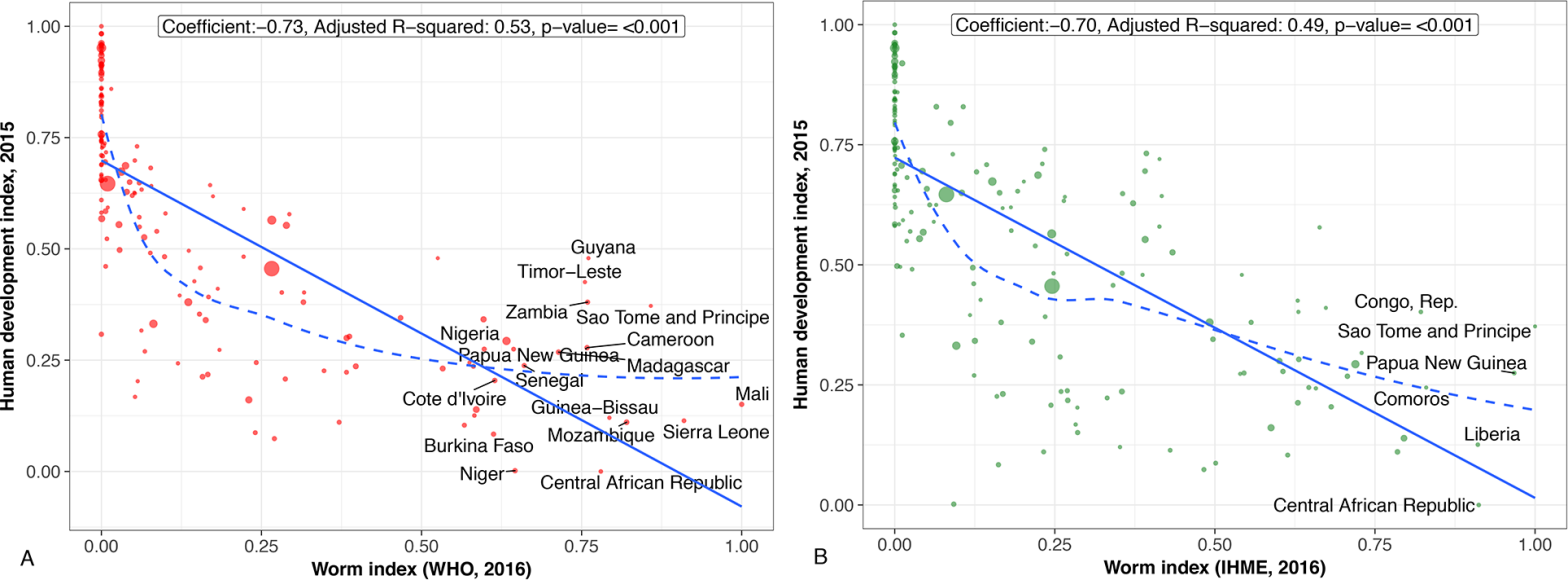
**A.** Regression plot between worm index and human development index. Worm indices calculated using WHO 2016 data. **B.** Regression plot between worm index and human development index. Worm indices calculated using GBD 2016 data. (Scaled to 0–1).

**Fig 2:**
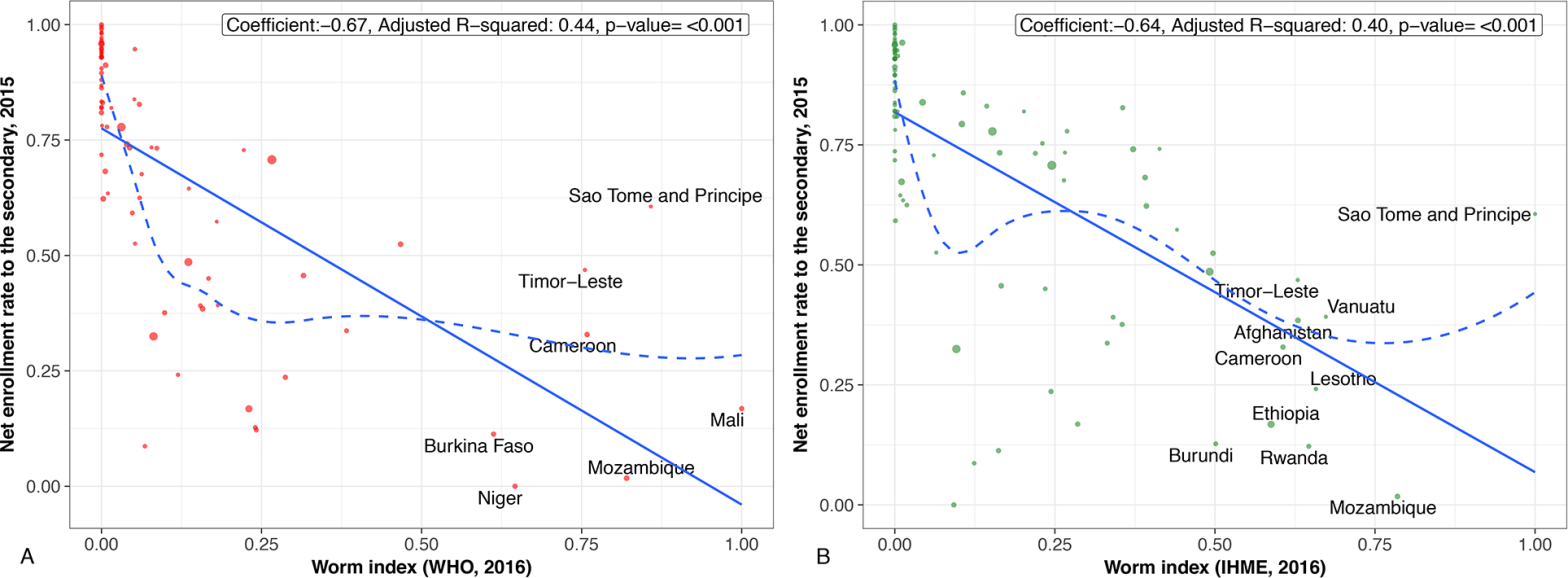
**A.** Regression plot between worm index and net enrollment rate, secondary. Worm indices calculated using WHO 2016 data. **B.** Regression plot between worm index and net enrollment rate, secondary. Worm indices calculated using GBD 2016 data. (Scaled to 0–1).

**Fig 3:**
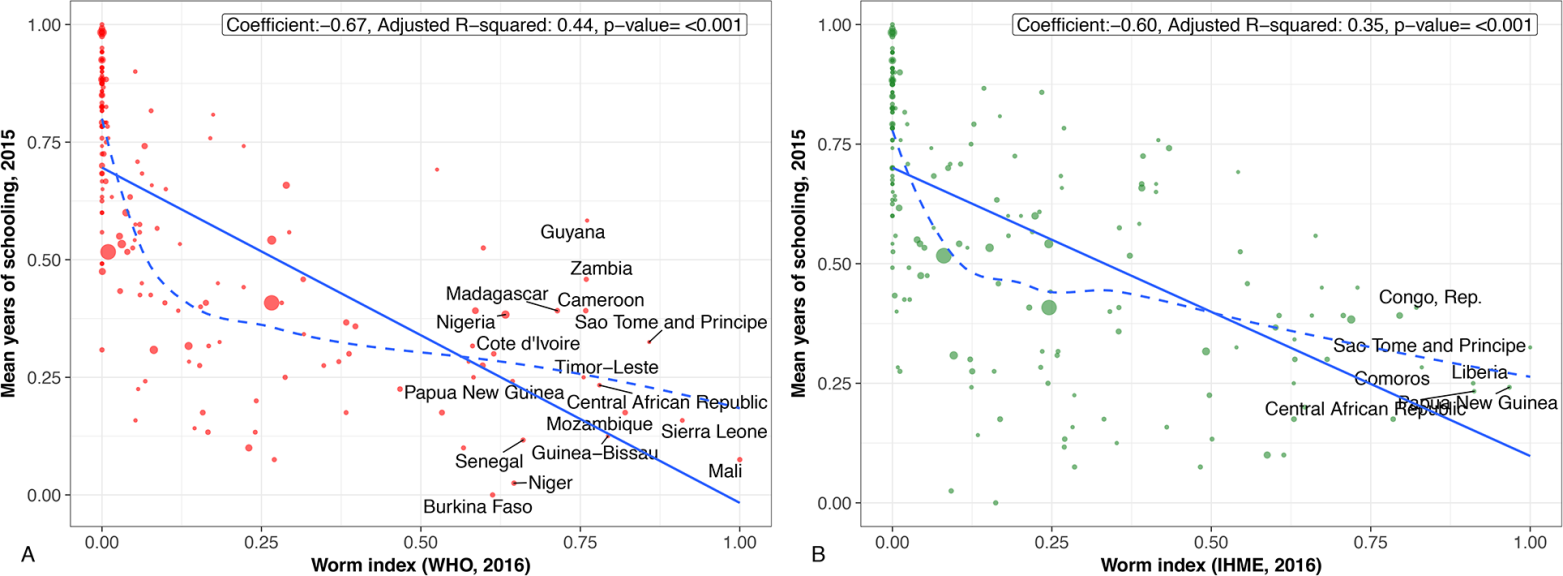
**A.** Regression plot between worm index and mean years of schooling. Worm indices calculated using WHO 2016 data. **B.** Regression plot between worm index and mean years of schooling. Worm indices calculated using GBD 2016 data. (Scaled to 0–1).

**Fig 4:**
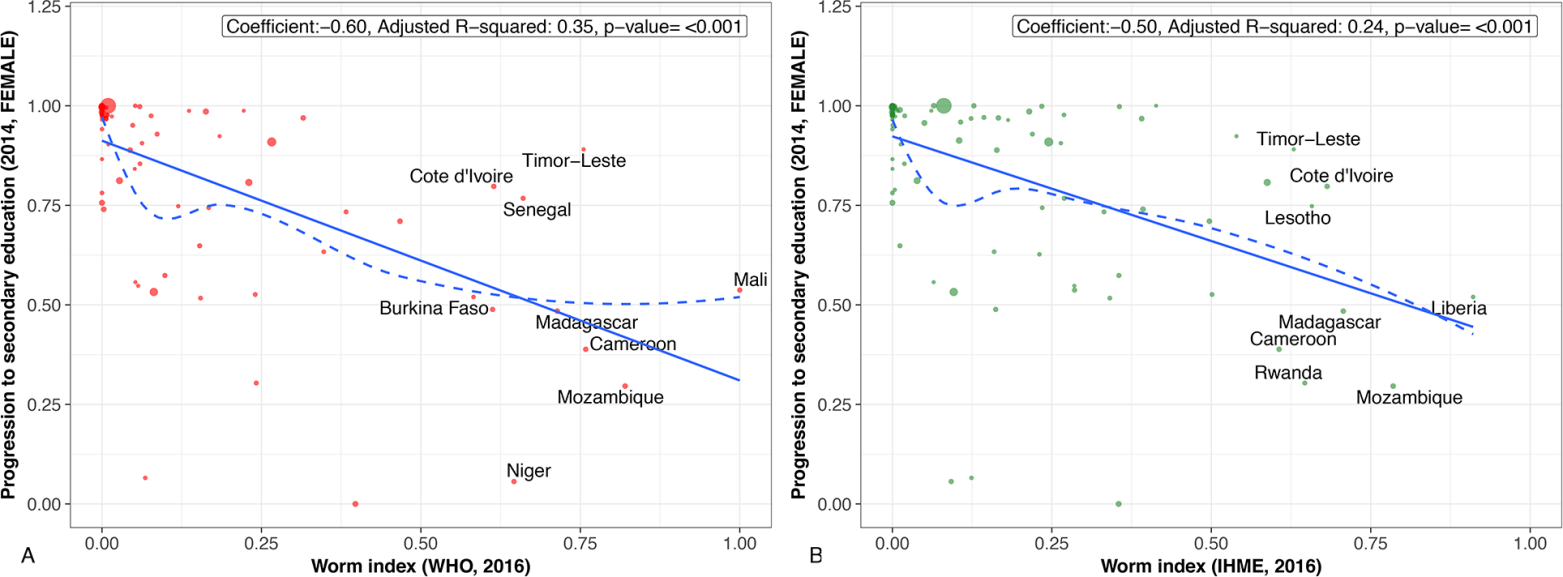
**A.** Regression plot between worm index progression to the secondary education, female. Worm indices calculated using WHO 2016 data. **B.** Regression plot between worm index progression to the secondary education, female. Worm indices calculated using GBD 2016 data. (Scaled to 0–1).

Figs 1A–4B present regression plots between worm index and selected outcome measures. The smoothed curves are obtained by Locally Weighted Scatterplot Smoothing (LOWESS). Overall, both WHO and GBD data confirm earlier findings of a negative association between worm indices and HDI, as well as educational attainment. R-square functions for each of the figures indicate that the approximately 50% variation in HDI measure is explained by worm indices alone calculated from WHO and GBD data (about 25%–45% variation in all education measures explained by worm indices alone calculated from WHO and GBD data). However, the smoothed curve for those countries with worm indices between 0 and 0.25 are concave-up. In other words, for many of the associations, worm indices may exert their greatest effects on human development and education indicators in countries with lower overall prevalence rates of parasitic worms or with lower numbers of children and adults who require MDA for their intestinal helminth infections, schistosomiasis, and LF. Generally speaking, such countries represent middle-income or low-income countries at the higher end.

Table 3 displays the world’s top 30 countries with the highest worm indices, along with their HDI rankings. Most of these countries, calculated by both WHO PCT and GBD data, are found also low-ranking groups in HDI. Nineteen of the top 30 countries appear on both ranking lists calculated from WHO and GBD data.

**Table 3:** Worm index rankings by country along with HDI.

Rank (WHO)	Country	HDI ranking	Rank (GBD)	Country	HDI ranking
1	Mali	175	1	Sao Tome and Principe	142
2	Sierra Leone	179	2	Papua New Guinea	154
3	Sao Tome and Principe	142	3	Central African Republic	188
4	Mozambique	181	4	Liberia	177
5	Guinea-Bissau	178	5	Comoros	160
6	Central African Republic	188	6	Congo, Rep.	135
7	Guyana	127	7	Congo, Dem. Rep.	176
8	Zambia	139	8	Mozambique	181
9	Cameroon	153	9	Swaziland	148
10	Timor-Leste	133	10	Nigeria	152
11	Madagascar	158	11	Madagascar	158
12	Senegal	162	12	Ivory Coast (Cote d'Ivoire)	171
13	Niger	187	13	Vanuatu	134
14	Papua New Guinea	154	14	Gabon	109
15	Nigeria	152	15	Lesotho	160
16	Ivory Coast(Cote d'Ivoire)	171	16	Rwanda	159
17	Burkina Faso	185	17	Angola	150
18	Zimbabwe	154	18	Equatorial Guinea	135
19	Myanmar	145	19	Afghanistan	169
20	Congo, Dem. Rep.	176	20	Timor-Leste	133
21	Liberia	177	21	Guinea	183
22	Haiti	163	22	Cameroon	153
23	Comoros	160	23	Tanzania	151
24	Guinea	183	24	Ethiopia	169
25	Sudan	165	25	Zimbabwe	174
26	Micronesia, Fed. Sts.	127	26	Micronesia, Fed.Sts	127
27	Nepal	144	27	Solomon Islands	156
28	Uganda	163	28	Burundi	184
29	Angola	150	29	Nepal	144
30	Tanzania	151	30	Bangladesh	139

Abbreviations: HDI, human development index; GBD, Global Burden of Disease.

In addition, we looked at the association between worm indices and HDI separated by 6 regions of the world defined by the WB (East Asia and Pacific, Europe and Central Asia, Latin America and Caribbean, Middle East and North Africa, South Asia, and sub-Saharan Africa). With WHO data, Latin America and Caribbean region shows the greatest decrease of SD unit in HDI (−0.707) change associated with one SD increase in worm index, followed by Europe and Central Asia (−0.674), sub-Saharan Africa (−0.525), and East Asia and Pacific (−0.59). In contrast, South Asia and Middle East and North Africa regions were not statistically significant. For the GBD data, all regions are statistically significant results. South Asia shows the greatest decrease of SD unit in HDI (−0.742), followed by Europe and Central Asia (−0.716), Latin America and Caribbean (−0.639), East Asia and Pacific (−0.634), Middle East and North Africa (−0.627), and sub-Saharan Africa (−0.447).

## Discussion

In summary, utilizing 2 different worm indices data available, our findings showed negative associations between helminthic neglected tropical diseases (NTDs) and both human development and educational indicators for children globally. The negative links to HDI for all 191 nations globally extend previous information provided for the 25 largest nations and OIC countries, respectively [1,2], while our new findings—the negative associations with educational development, including school enrollment rate, mean years of schooling, and school persistence—generate a number of potentially interesting hypotheses for further testing.

The negative association of worm infections with child intelligence and educational attainment was noted almost 100 years ago among school children infected with hookworm in rural Alabama [8]. It’s of interest to note that a recent study found that hookworm remains endemic to rural Alabama and possibly elsewhere in the American South [9]. Throughout the decade of the 1990s and into the early 2000s, it was noted that intestinal worm infections and schistosomiasis exert adverse effects on the cognitive function of school-aged children in poor areas of Africa, Asia, and the Americas [10–15], and these findings provided a basis to recommend frequent and periodic deworming, especially in schools [16, 17], as well as to the adoption of a World Health Assembly resolution for global scale-up of MDA [18]. Since then, school-based deworming has been integrated with other NTD control measures, including MDA for LF and onchocerciasis through a package of interventions [19], which, according to WHO, are now reaching hundreds of millions of children globally [20].

More recently, Kremer and Miguel have conducted randomized control trials (RCTs) to show that deworming has strong effects on improving school participation and reducing absenteeism in Kenya [21]. The low cost of deworming with donated anthelminthic drugs suggests that this approach represents a highly cost-effective intervention [21, 22]. Indeed, a retrospective economic analysis from the American South further suggests that these educational improvements translate to a beneficial economic impact [23], findings also reproduced in resource-poor countries [24].

Countering these findings, a systematic review and meta-analysis based on RCTs has called into question the benefits of deworming on child nutrition and school performance [25], leading to an interesting discussion (sometimes referred to as “worm wars”) on how to best shape global policy on this front [26–28]. Could our finding reported here—that the impact of worm indices on education and development appears stronger in countries with worm indices less than 0.25—be relevant to the worm wars? For instance, nations with the highest worm indices are typically the world’s lowest-income nations, which are also beset by high rates of pediatric malaria, lower respiratory infections, and diarrheal diseases. In such nations, it might be more difficult to demonstrate effects of deworming in children simultaneously affected by serious coinfections. Alternatively, in nations with lower worm indices and higher levels of human development, it might be easier to demonstrate the impact of deworming due to the absence or lower rates of these coinfections. In this regard, it’s interesting to note that better-off regions of the world, such as Latin America and the Caribbean, tended to show a greater decrease in HDI due to worm index compared to sub-Saharan Africa.

Overall, the results here indicate a strong negative association between high endemic worm burdens from intestinal helminth infections, schistosomiasis, and LF and negative educational attainment. In terms of interpretation, one has to be careful, however, of not reading too much into the bivariate associations without further multivariate analysis and appropriate statistical modeling, which can help us better understand the extent to which there is reverse causality (high-HDI countries tend to have lower worms) or the extent to which there are other “omitted variables”—countries that have low HDI and high-worm indices, for example, may also tend to be those with bad governance, which makes deworming ineffective in them.

Nevertheless, our findings indicate an urgent need to better explore mechanisms behind worm-associated educational deficits and for studies on mechanisms to confirm whether the worms exert these effects directly, possibly through malnutrition, or whether worms are “biomarkers” indicative of severe poverty or other negative aspects of human development. The general finding of potential greater negative educational effects on female children is also of interest for future research. Further studies are underway to better identify the links between high-worm indices and poor education and to identify potential mechanisms. Finally, in terms of data collection and reporting, despite the similar results between WHO and GBD data, sometimes discrepant results suggest that there is pressing need for more extensive and coherent high-quality data.

Our findings reported here also have potential implications for public policy. Confirmation of our identified links between human helminth infection and development highlight key roles for the WB and international ministries of finance to prioritize global deworming as a potentially important tool for human capital accumulation and global economic development. Linking human helminth infection to education, especially the education of girls and women, could shape new public policies around global deworming as essential to the United Nations Girls Education Initiative (UNGEI), which was launched in the same year as the UN Millennium Development Goals. Similarly, the emphasis on deworming in girls and women as endorsed by the WHO’s 2017 Bellagio Declaration for NTDs would align well with our findings.

In summary, our results here generate some key and provocative hypotheses that take global deworming beyond the health sector by linking such activities to international development and education. Potentially, our findings also highlight research into new anthelminthic drugs, diagnostics, and vaccines as novel technologies for advancing educational attainment and economic development.

## Supporting information

S1 Table Worm index rankings by country. (DOCX)
