# Supplementary material for: Extending the global worm index and its links to human development and child education
Source: PLoS Negl Trop Dis. 2018 Jun 21;12(6):e0006322. doi: 10.1371/journal.pntd.0006322 (PMC6013015; doi:10.1371/journal.pntd.0006322)
Supplement: S1 Table — (DOCX) [file pntd.0006322.s001.docx]

S1 Table

| **S1 Table. Worm index rankings by country** | | | |
| --- | --- | --- | --- |
| **Rank** | **Country** | **Rank (GBD)** | **Country** |
| **(WHO)** |  |  |  |
| 1 | Mali | 1 | Sao Tome and Principe |
| 2 | Sierra Leone | 2 | Papua New Guinea |
| 3 | Sao Tome and Principe | 3 | Central African Republic |
| 4 | Mozambique | 4 | Liberia |
| 5 | Guinea-Bissau | 5 | Comoros |
| 6 | Central African Republic | 6 | Congo, Rep. |
| 7 | Guyana | 7 | Congo, Dem. Rep. |
| 8 | Zambia | 8 | Mozambique |
| 9 | Cameroon | 9 | Swaziland |
| 10 | Timor-Leste | 10 | Nigeria |
| 11 | Madagascar | 11 | Madagascar |
| 12 | Senegal | 12 | Ivory Coast (Cote d'Ivoire) |
| 13 | Niger | 13 | Vanuatu |
| 14 | Papua New Guinea | 14 | Gabon |
| 15 | Nigeria | 15 | Lesotho |
| 16 | Ivory Coast | 16 | Rwanda |
| 17 | Burkina Faso | 17 | Angola |
| 18 | Zimbabwe | 18 | Equatorial Guinea |
| 19 | Myanmar | 19 | Afghanistan |
| 20 | Congo, Dem. Rep. | 20 | Timor-Leste |
| 21 | Liberia | 21 | Guinea |
| 22 | Haiti | 22 | Cameroon |
| 23 | Comoros | 23 | Tanzania |
| 24 | Guinea | 24 | Ethiopia |
| 25 | Sudan | 25 | Zimbabwe |
| 26 | Micronesia, Fed. Sts. | 26 | Micronesia, Fed.Sts |
| 27 | Nepal | 27 | Solomon Islands |
| 28 | Uganda | 28 | Burundi |
| 29 | Angola | 29 | Nepal |
| 30 | Tanzania | 30 | Bangladesh |
| 31 | Benin | 31 | Somalia |
| 32 | South Sudan | 32 | Chad |
| 33 | Togo | 33 | Marshall Islands |
| 34 | Equatorial Guinea | 34 | South Africa |
| 35 | Ghana | 35 | Sierra Leone |
| 36 | Gabon | 36 | Fiji |
| 37 | Philippines | 37 | Seychelles |
| 38 | Malawi | 38 | Botswana |
| 39 | Congo, Rep. | 39 | Malaysia |
| 40 | Chad | 40 | Philippines |
| 41 | Indonesia | 41 | Venezuela, RB |
| 42 | India | 42 | Guyana |
| 43 | Rwanda | 43 | Colombia |
| 44 | Burundi | 44 | Ecuador |
| 45 | Ethiopia | 45 | Guatemala |
| 46 | Samoa | 46 | Uganda |
| 47 | Namibia | 47 | Guinea-Bissau |
| 48 | Solomon Islands | 48 | Honduras |
| 49 | Vanuatu | 49 | Benin |
| 50 | Marshall Islands | 50 | Mali |
| 51 | Somalia | 51 | Djibouti |
| 52 | Tonga | 52 | Gambia, The |
| 53 | Fiji | 53 | Yemen, Rep. |
| 54 | Lao PDR | 54 | Senegal |
| 55 | Yemen, Rep. | 55 | Kyrgyz Republic |
| 56 | Kenya | 56 | St. Lucia |
| 57 | Afghanistan | 57 | Jamaica |
| 58 | Honduras | 58 | Haiti |
| 59 | Cambodia | 59 | Syrian Arab Republic |
| 60 | Bhutan | 60 | Namibia |
| 61 | Cabo Verde | 61 | American Samoa |
| 62 | Bangladesh | 62 | India |
| 63 | Kiribati | 63 | Indonesia |
| 64 | Lesotho | 64 | Malawi |
| 65 | Botswana | 65 | Lao PDR |
| 66 | Guatemala | 66 | Kazakhstan |
| 67 | Bolivia | 67 | South Sudan |
| 68 | Pakistan | 68 | Costa Rica |
| 69 | St. Lucia | 69 | Mexico |
| 70 | Azerbaijan | 70 | Bolivia |
| 71 | Nicaragua | 71 | Kenya |
| 72 | Mauritania | 72 | Grenada |
| 73 | South Africa | 73 | Jordan |
| 74 | Jamaica | 74 | Paraguay |
| 75 | Swaziland | 75 | St. Vincent and the Grenadines |
| 76 | El Salvador | 76 | Sudan |
| 77 | Ecuador | 77 | Tonga |
| 78 | Paraguay | 78 | Ghana |
| 79 | Djibouti | 79 | Peru |
| 80 | Panama | 80 | Burkina Faso |
| 81 | Gambia, The | 81 | Togo |
| 82 | Suriname | 82 | Brazil |
| 83 | Georgia | 83 | Cuba |
| 84 | Dominica | 84 | Bhutan |
| 85 | Dominican Republic | 85 | Sri Lanka |
| 86 | Peru | 86 | Myanmar |
| 87 | Colombia | 87 | Mauritania |
| 88 | Mexico | 88 | Tajikistan |
| 89 | Brazil | 89 | Morocco |
| 90 | Iraq | 90 | Kiribati |
| 91 | Vietnam | 91 | Chile |
| 92 | Brunei Darussalam | 92 | Thailand |
| 93 | Belize | 93 | Pakistan |
| 94 | China | 94 | Niger |
| 95 | Kyrgyz Republic | 95 | Panama |
| 96 | Trinidad and Tobago | 96 | Argentina |
| 97 | Armenia | 97 | China |
| 98 | Tajikistan | 98 | Saudi Arabia |
| 99 | Uzbekistan | 99 | Suriname |
| 100 | Venezuela, RB | 100 | Samoa |
